# Biological Activity, Hepatotoxicity, and Structure-Activity Relationship of Kavalactones and Flavokavins, the Two Main Bioactive Components in Kava (*Piper methysticum*)

**DOI:** 10.1155/2021/6851798

**Published:** 2021-08-20

**Authors:** Yingli Wang, Chao Su, Bo Zhang, Yang Niu, Ruru Ren, Xiaojun Zhao, Lingling Yang, Wannian Zhang, Xueqin Ma

**Affiliations:** Department of Pharmaceutical Analysis, School of Pharmacy, Key Laboratory of Hui Ethnic Medicine Modernization, Ministry of Education, Ningxia Medical University, 1160 Shenli Street, Yinchuan 750004, China

## Abstract

Kava (*Piper methysticum* Forst) is a popular and favorable edible medicinal herb which was traditionally used to prepare a nonfermented beverage with relaxant beneficial for both social and recreational purposes. Numerous studies conducted on kava have confirmed the presence of kavalactones and flavokawains, two major groups of bioactive ingredients, in this miraculous natural plant. Expectedly, both kavalactone and flavokawain components exhibited potent antianxiety and anticancer activities, and their structure-activity relationships were also revealed. However, dozens of clinical data revealed the hepatotoxicity effect which is indirectly or directly associated with kava consumption, and most of the evidence currently seems to point the compounds of flavokawains in kava were responsible. Therefore, our aim is to conduct a systematic review of kavalactones and flavokawains in kava including their biological activities, structure-activity relationships, and toxicities, and as a result of our systematic investigations, suggestions on kava and its compounds are supplied for future research.

## 1. Introduction

*Piper methysticum* Forst, popularly known as kava, is an edible and medicinal plant of shrub which has history of more than 2000 years. Given the purposes for religious occasions, medicinal purposes, and social gatherings [1–3], kava is particularly important for the indigenous people of the Pacific Rim and the Hawaiian Islands [4]. In the daily life of the South Pacific island people, the water infusion of kava root was used as a traditional beverage since ancient times for its sedative and calming effects, such as soothing the nerves, inducing relaxation and sleep, counteracting fatigue, and reducing weight [5, 6], and the dietary supplements of kava were easily obtained in some health food stores [7]. Similarly, in the Western world, pharmaceutical preparations of this herb were commonly applied for the treatment of anxiety disorders.

However, there was compelling evidence that kava consumption was related to some toxicities which led to its restriction or warning in many countries since 2002 [8, 9]. Several studies have reported a series of adverse health effects including kava dermopathy [10], hepatotoxicity [11, 12], and the disruption of cognition [13, 14] which were associated with kava consumption. Among those, kava hepatotoxicity was the most concerning adverse effect of kava consumption.

Although several of the published reviews have summarized the pharmacology, safety profiles associated with kava [3, 9, 15], however, over the past decades, dozens of studies which focused on the chemical constituents and biological activities of kava have been disclosed and some possible mechanisms of action have also been explored. Also, we found some scientific gaps still existed in the specific mechanism of its anticancer effect, as well as the detailed pathogenetic factors of kava hepatotoxicity, especially the flavokawain components which were believed to b responsible for the hepatotoxicity. Furthermore, the clinical evidence for the treatment of generalized anxiety disorder (GAD) and the responsibility components was also not clear. The aim of this paper is to give a full-scale profile for the research of kava kavalactones and flavokavins, focusing on their available scientific information including the chemical structures, structure-activity relationships, biological activities, and toxicities.

## 2. Chemical Constituents

Until now, more than 56 constituents have been isolated and identified from *P. methysticum*. These can be assigned to two main classes, kavalactones and flavokavins. The details of each type of compounds are summarized below.

### 2.1. Kavalactones

**Figure 1:**
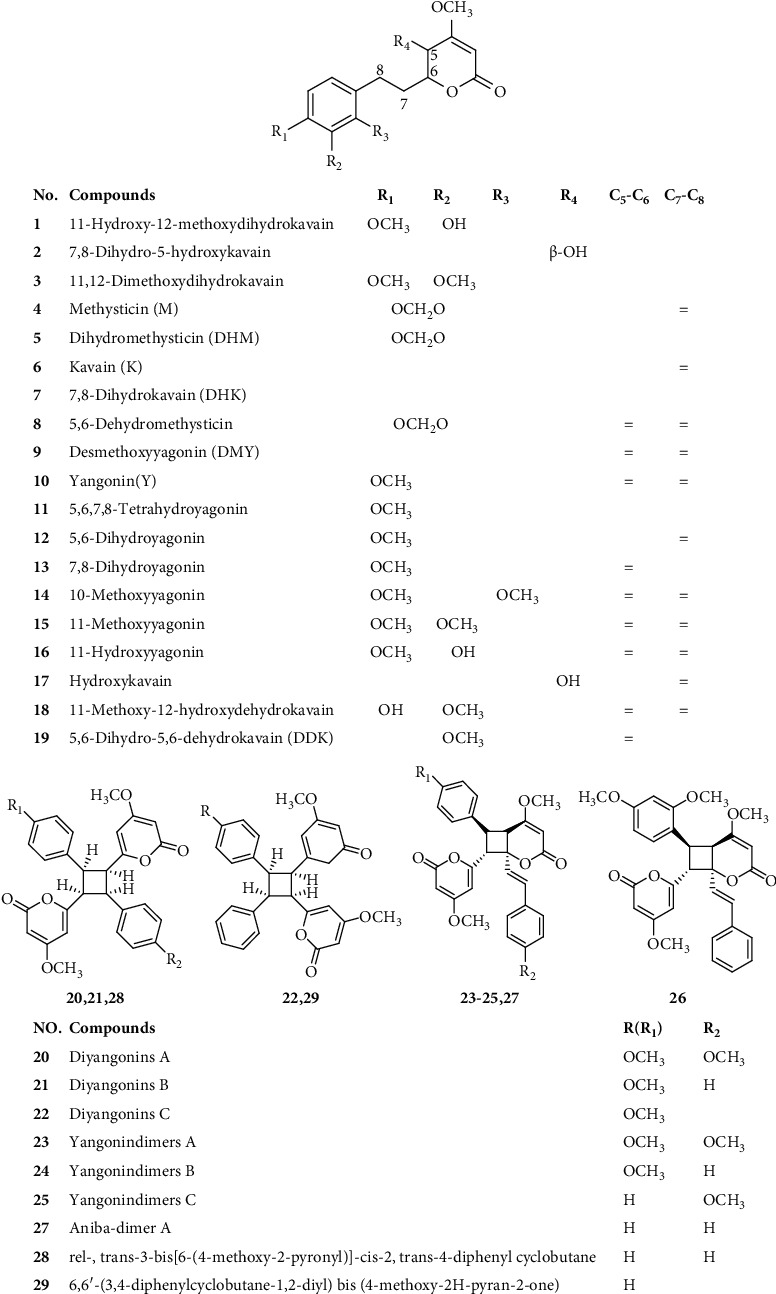
Chemical structures of compounds **1**–**29**.

**Figure 2:**
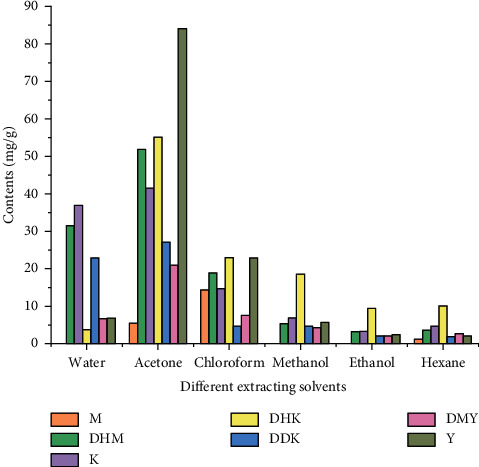
Content of seven major kavalactones in kava with different extracting solvents (mg/g extract).

Kavalactones belong to lipophilic lactones with an *α*-pyrone skeleton typically 4-methoxy-2-pyrones, and aromatic stiryl or phenylethyl was substituted at the 6-position [16]. At present, 29 kavalactones, shown in Figure 1, have been isolated and identified. Kavalactones can be extracted from the rhizomes, roots, and root stems of the plant [9]. By employing gas chromatography-mass spectrometer (GC-MS) combined with high-performance liquid chromatography (HPLC) techniques, the extracting efficacies of different solvents (water, acetone, chloroform, methanol, ethanol, and hexane) on the contents of kavalactone constituents were determined [5], as Figure 2 shows. Seven major kavalactones, namely, methysticin (**4**), dihydromethysticin (**5**), kavain (**6**), 7, 8-dihydrokavain (**7**), desmethoxyyagonin (**9**), yangonin (**10**), and 5,6-dihydro-5,6-dehydrokavain (**19**), were obtained from the kava roots. It was found that acetone was the most effective solvent in terms of yield and quantities of kavalactone compounds obtained. The contents of seven major kavalactones including methysticin dihydromethysticin, kavain, 7, 8-dihydrokavain, desmethoxyyagonin, yongonin, and 5,6-dihydro-5,6-dehydrokavain were 1.2–14.4 mg/g, 3.2–51.9 mg/g, 3.3–41.5 mg/g 3.8–55.1 mg/g, 2.1–21 mg/g, 2.1–84.1 mg/g, and 1.9–27.1 mg/g, respectively [5].

A series of kavalactone dimers were also isolated and identified via extensive phytochemical investigation on the roots of kava [17–19]. By using classical chromatographic separation methods combined with spectrum identification techniques, a series of novel dimeric kavalactones, namely, diyangonins A (**20**), diyangonins B (**21**), diyangonins C (**22**), yangonindimers A (**23**), yangonindimers B (**24**), yangonindimers C (**25**), kavalactone A (**26**), aniba-dimer A (**27**), rel-, trans-3-bis[6-(4-methoxy-2-pyronyl)]-cis-2, trans -4-diphenyl cyclobutane (**28**), and 6,6′-(3,4-diphenylcycl-obutane-1,2-diyl) bis (4-methoxy-2H-pyran-2-one) (**29**), were isolated and elucidated from kava [17–19]. The chemical structures of compounds **20**–**29** are listed in Figure 1.

### 2.2. Flavokavins

**Figure 3:**
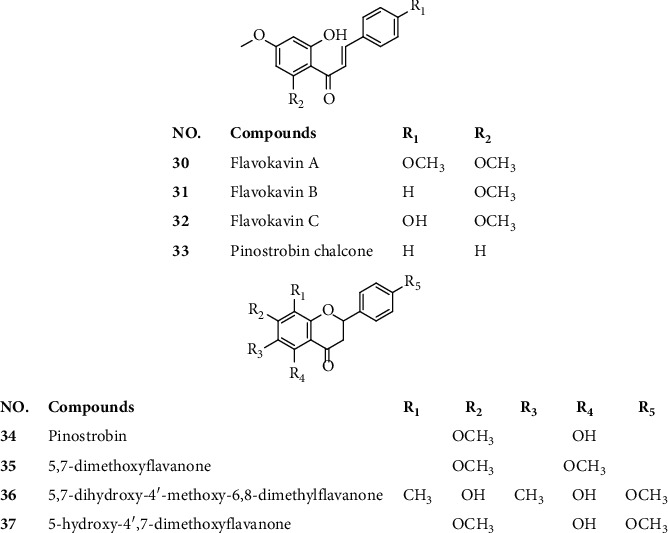
Chemical structures of compounds **30**–**37**.

The first three dihydrochalcones, namely, flavokavin A (**30**), flavokavin B (**31**), and flavokavin C (**32**), were isolated from the roots of kava by using the high-performance thin-layer chromatography (HPTLC) method [20]; followed by the pinostrobin chalcone (**33**), which was detected in kava roots for the first time by employing GC-MS and HPLC analysis [5]. Recently, two new flavanones, namely, pinostrobin (**34**) and 5,7- dimethoxyflavanone (**35**) [21], along with 5,7-dihydroxy-4′-methoxy- 6,8-dimethylflavanone (matteucinol,**36**) and 5-hydroxy- 4′,7- dimethoxyflavanone (**37**) have been obtained via column chromatography (CC) and HPLC methods [5, 22]. The chemical structures of these flavanones are listed in Figure 3.

## 3. Biological Activities

**Figure 4:**
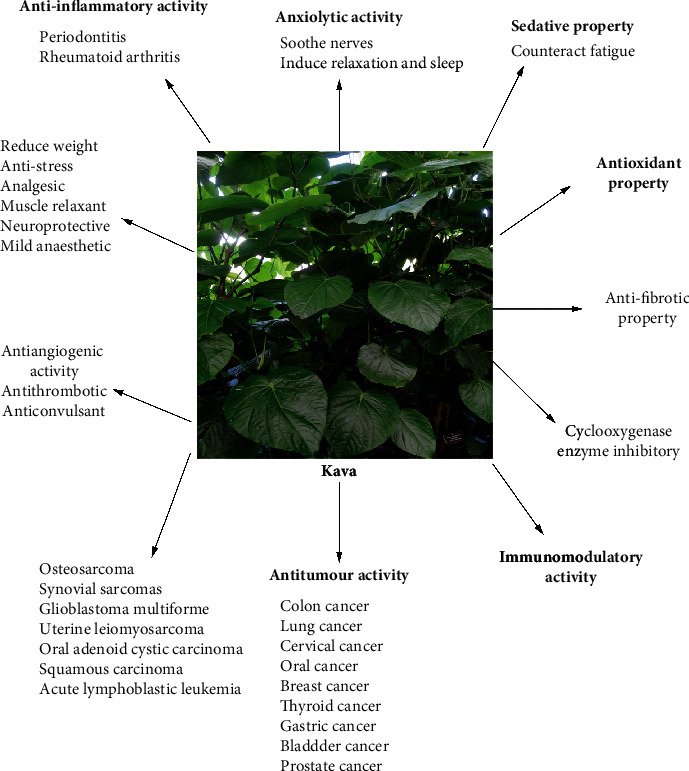
The pharmacological activities of kava.

**Figure 5:**
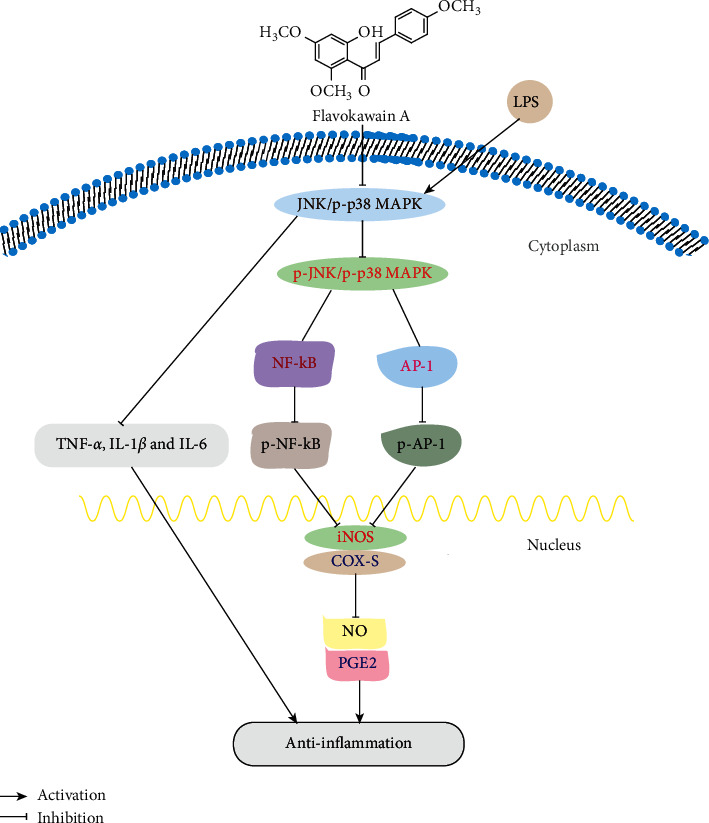
The proposed model of the FKA-mediated anti-inflammation via nuclear factor-*κ*B (NF-*κ*B) blockade and AP-1 activation in RAW 264.7 macrophages.

**Figure 6:**
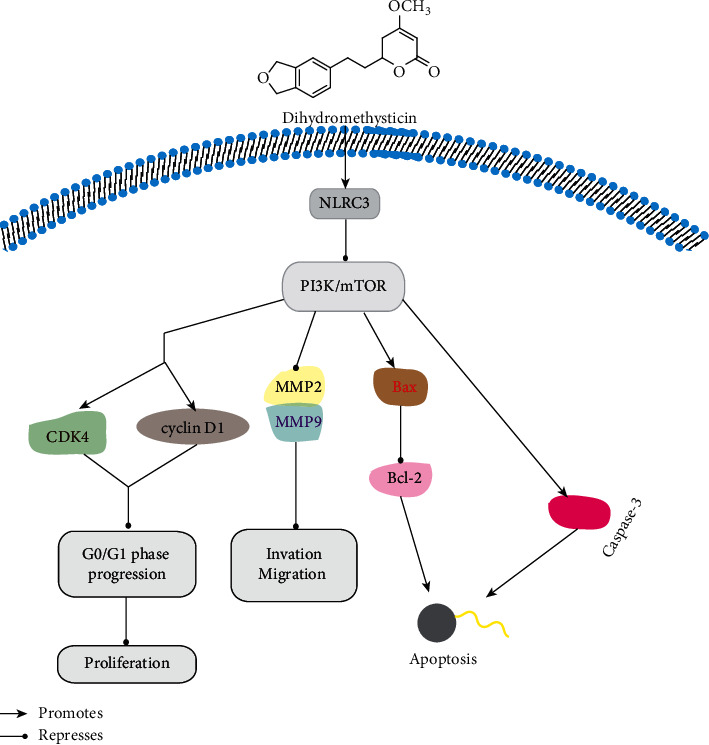
Proposed diagrams of DHM-induced *G*0/*G*1 phase arrest and apoptosis through phosphoinositide 3-kinase (PI3K)/nucleotide-oligomerization domain-like receptor subfamily C3 (NLRC3) signaling pathway inhibition in colorectal cancer cells.

Various uses and pharmacological properties of the isolated kavalactones and flavokavins from the rhizomes and roots of kava have been reported (Table 1; Figure 4). Lately, a published review has summarized the anti-inflammatory activity, neurological functions, and anticancer property of kava and its components [58]. To avoid repetition and exhibit our innovation, we supplied the details about the abovementioned activities of kavalactones and flavokavins, including the *in vitro* cell models and *in vivo* animal models, the methods of the experiments, the major findings, and the possible mechanisms, for example, the anti-inflammatory mechanisms of FKA, as Figure 5 shows, and the anticancer mechanisms of DHM, as Figure 6 described. All of them are exhibited in Table 1 and Figures 4–6.

## 4. Kava Hepatotoxicity

Kava became a well-known edible medicinal herb not only for its excellent activity but also for its controversy toxicity, and kava hepatotoxicity was the most concerning adverse effect of kava consumption [11]. Since the first case of kava hepatotoxicity was reported by in 1998 [59], more than 100 cases of severe liver injury following kava exposure have been identified all over the world. However, many of which were uncertain whether kava was responsible or it was caused by the other possible pathogenetic factors which were overlooked in reported cases of kava hepatotoxicity. For example, kava consumption involved concomitant ingestion of other agents with potential hepatotoxicity including other medications and/or alcohol [9]. Furthermore, the number of cases might be overstated as the types of liver injury noted include necrosis, drug-induced hepatitis, and cholestatic hepatitis [3]. It was interesting to note that, in the South Pacific, the adverse effect of liver damage was virtually absent during kava consumption. Cytochrome P450 2D6 (CYP2D6), an important enzyme which was necessary during drug metabolism, could also mediate the drug-drug interactions and, thus, might be responsible [60]. During the past years, suggestions and discussions have revealed the possible pathogenetic factors leading to the development of kava hepatotoxicity [11], and the details are given in the following.

### 4.1. Different Sources and Parts of Kava for Practical Applications

Concerning the early history of kava, the lack of standard kava raw material might be the major factor, at least in some cases [61]. The different parts of the kava plant possessed different compounds, which showed different kava raw materials might contain different contents of the toxic constituents and then influenced the function of liver [11], for example, the substandard kava cultivars, different growth ages, using stem peelings replaced kava toots, rhizomes, or aerial parts of the kava plants (contains toxic alkaloids), and contamination of aflatoxicosis or other mould hepatotoxins [61]. Therefore, the botanical characteristics of the plant and the harvesting and storage conditions might be involved in the development of hepatotoxicity and triggering idiosyncratic reaction [11, 62].

### 4.2. Different Solvents Used for Kava Extraction

The next concern was whether the liver is damaged following kava consumption due to the solvent used for kava extract preparation or not [11]. Because the kavalactone and flavokavins contained in kava possessed different polarities, employing ethanol and acetone as solvents for the extraction of kava generally yielded high contents of kavalactone. As the higher portions of kavalactones was proved to be usually associated with liver failure, thus, using acetone as the extracted solvent might concentrate the toxic components. However, the results came from the World Health Organization (WHO) study which reported five live injury cases which were associated with the aqueous extracts of kava. Therefore, the solvent itself fails to involve in the overall pathogenesis of kava hepatotoxicity [11, 60].

### 4.3. Comedication, Overdose, and Prolonged Use

In many kava hepatotoxicity cases, other concurrent medications being taken by patients also existed; thus, it was uncertain whether the hepatotoxic reaction was initiated by kava itself or other drugs. Theoretically, the metabolic process of complicated drugs might be altered in some especially cases, and even the components themselves lacking evidences of hepatotoxicity might also exert hepatotoxic effects. Therefore, at least in some clinical cases, the interaction between kava and drug might be a potential factor for the hepatotoxicity [11]. Furthermore, prolonged kava treatment as well as overdose of kavalactones should not be overlooked [63, 64]. It was disclosed that nonadherence to medication was a common matter but not unique for kava treatment. However, at present, there are no studies that focus on the abovementioned subjects. Therefore, it was not available to answer the issue of kava hepatotoxicity that might be related with prolonged and overdose, and further experimental assessment was necessary.

### 4.4. Toxic Constituents, Metabolites, and Contaminations

Other unknown toxic components, the contaminations derived from various kava extracts, and storage process could not be excluded for the moment, for example, the piperidine alkaloid pipermethystine in aerial parts of the kava plants, the contamination of aflatoxicosis or other *mould hepatotoxins* [11, 65] during long time, and improper storage. It was proved that the alkaloid pipermethystine could induce liver cell death by glutathione (GSH) depletion and modulate MAPK and NF-*κ*B signaling pathways *in vitro* [66]. However, other *in vivo* experimental animal studies obtained the converse results, which failed to cause any liver damage during alkaloid pipermethystine treatment. Therefore, it was uncertain that pipermethystine had the responsibility between kava and hepatotoxicity [61, 66, 67]. In addition, FKB has been considered as a possible pathogenetic factor for human kava hepatotoxicity [61, 68]. It could induce cell apoptosis in hepatoblastoma (HepG2) (LD_50_ = 15.3 ± 0.2 *μ*M) and L-02 (LD_50_ = 32 *μ*M) cells via inducing oxidative stress, reducing the depletion of glutathione and inhibiting the I-*κ*B kinase (IKK) activity *in vitro* [69]. FKB, meanwhile, induced hepatic damage by inhibiting NF-*κ*B transcriptional activity *in vivo* [61, 68, 69]. Furthermore, kava hepatotoxicity also involved concomitant ingestion of other agents such as alcohol; thus, the metabolic interactions of kava with alcohol might also be a possible mechanism [70].

## 5. The Investigation of the Structure-Activity Relationship (SAR)

The structure-activity relationship study is a widely used and well-established method for the early drug discovery stage. The structural-based activity information was usually employed to screen for or optimize compounds to achieve drug-like properties [71]. Kavalactones and flavokawains possessed the unique pharmacological effects including the efficacy and side effects, which were all directly related to their structures [72]. Recently, different synthetic approaches of kavalactones, as well as the key biosynthetic enzymes of the kavalactone and flavokavain, were reported [73, 74]. However, the difficulty of biosynthetic and chemical synthesis hindered the therapeutic use of kavalactones and flavokawains in both laboratory experiments and clinical trials [74]. In order to improve the efficacy and pharmaceutical properties of kavalactones and flavokavins, further medicinal chemistry optimization is needed.

### 5.1. Kavalactone Analogues

**Figure 7:**
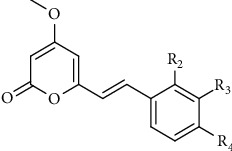
The structure modification of kavalactone.

Lately, it was explored that kavalactone analogues exhibited *in vitro* anthelmintic activities against *Haemonchus contortus* larvae [75]. Through the chemical modifications of 2- ,3-, and 4-substituent on the pendant aryl ring (Figure 7), two kavalactones (yangonin and desmethoxyyangonin) and 17 analogues were synthesized. Among these analogues, compounds with 4-trifluoromethoxy, 4-phenoxy, 4-difluoromethoxy, and 4-N-morpholine substitutions showed convinced anthelmintic activities (1.9 *μ*M < IC_50_ < 8.9 *μ*M) which were superior to desmethoxyyangonin (IC_50_ = 37.1 *μ*M) and yangonin (IC_50_ = 15.0 *μ*M) and, thus, provided an opportunity for developing novel anthelmintic agents [75].

Besides kavalactone, kavain analogues were also designed and synthesized through chemical modifications. The results of pharmacodynamic tests showed that the synthesized compounds possessed anti-inflammation [25, 27, 28, 72, 76] and analgesic activities [77]. Kava-241, a kavain-derived compound, showed convinced efficacy in the prevention or treatment of advanced periodontal inflammation and related alveolar bone destruction *in vitro* and *in vivo* [27, 28] and, thus, might be a promising therapeutic agent against periodontal diseases in the future. Kav001, another kavain analogue, was designed and synthesized through optimizing the biological activity and structural physicochemical properties of kavain [24, 25]. Expectedly, kav001 displayed stronger analgesic activity than kavain [77].

### 5.2. Flavokawain Derivatives

**Figure 8:**
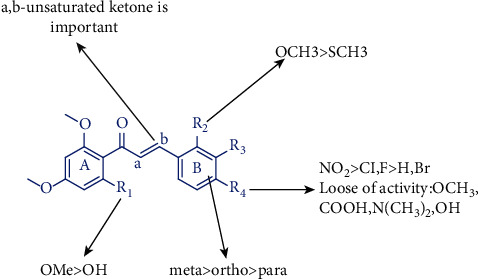
The structure-activity relationships of flavokawain.

Chalcones, an *α*,*β*-unsaturated ketone, was explored generally due to its simple chemistry structure, ease of synthesis, diversity of substituents, and wide range of biological activities [78, 79]. Flavokawain was a kind of chalcones which was widely occurring in plants [78]. Through chemical modifications of the A-ring (R_1_ site) and B-ring (R_2_, R_3_, and R_4_ site) (Figure 8), several flavokawain derivatives were designed, synthesized, and characterized. The anticancer properties of flavokawain in kava have been estimated due to the presence of the *α*, *β*-unsaturated ketone part through the structure-activity relationship studies of flavokawain derivatives [80]. The presence of electron-withdrawing and electron-donating groups could influence the effects of the *α*, *β*-unsaturated system and then cause the change of cytotoxicity [81]. Meanwhile, the presence of a hydroxyl group on the A-ring, rather than the B-ring, made the flavokawain derivatives more stable [80, 81]. Furthermore, effects of different functional groups were studied via substituent modification of the *ortho*, *meta,* and *para* positions on the B-ring. It was well established that the steric hindrance played a key role in the activity of flavokawain derivatives, which might exert cytotoxicity against cancer cell lines [82, 83]. The structure-activity relationship studies of flavokawain derivatives indicated that trimethoxy of the A-ring showed the most convinced cytotoxicity and selectivity, followed by the modification of the *meta* position on the B-ring and the substitution of halogen groups [82]. For example, (E)-1-(2′-hydroxy-4′,6′-dimethoxyphenyl)-3-(4-methylthio) phenyl) prop-2-ene-1-one (FLS), a flavokawain derivative, showed good selectivity against the breast cancer MCF-7 cell line [84].

## 6. Kava Metabolism

The pharmacokinetics and pharmacodynamics studies of kava in humans were carried out by means of experiments involving self-medication [85]. In humans, kavalactones as well as their metabolites were generally eliminated in the urine and feces, and the peak plasma levels usually occur around 2 h after ingestion, with a half-life of about 9 h. Orally administered kava water extracts were excreted mostly unchanged into urine [86]. The metabolism of kavain studied by the human liver cell-line Hep-G2 [87] or human serum and urine [85] disclosed the metabolites of kava including p-hydroxykavain, p-hydroxy-7,8-dihydrokavain, 5,6-dehydrokavain, 6-phenyl-5-hexen-2,4-dione [85], p-hydroxy-5,6-dehydrokavain, and 6-phenyl-3-hexen-2-one [88]. In rats, approximately 50% to 75% of kavalactones were excreted as glucuronide and sulphate conjugates in the urine and 15% was in the bile [89–91]. The most frequent metabolic pathways for kavalactones in humans and rats included hydroxylation of the C-12 in the aromatic ring, hydroxylation and cleavage of the lactone ring with subsequent dehydration, reduction of the 7,8-double bond, demethylation of the 4-methoxyl group, reduction of the double bond at carbons in positions 3 and 4 (to form a saturated pyrone ring system), and demethylation of the 4-methoxy group in the *α*-pyrone ring or of the 12-methoxy substituent in yangonin [89, 90, 92].

## 7. Conclusions and Future Perspectives

Kava is a magical plant composed of various constituents, and furthermore, it possessed anxiolytic relaxant effects in the treatment of anxiety disorders and also exhibited the potential activities in cancer prevention and therapy. Phytochemical investigations on kava plant have resulted in the isolation and identification of at least 56 compounds. Among them, kavalactones and dihydrochalcones were found to be the most widely studied chemical classes. In the last two decades, the separation and determination methods of kava extractions have gone through several technological innovations. So far, many new techniques were also developed for the qualitative and quantitative analysis of kava. However, more efficient and effective analytical methods are needed to determine the content of bioactive constituents and other unknown compounds on kava quality assessment due to the safety concerns of hepatotoxicity and other adverse effects [93, 94]. In summary, the possible pathogenic factors leading to the occurrence of kava hepatotoxicity were as follows: (1) the quality of kava raw material might be the major factor [61]; (2) concomitant ingestion of other drugs with potential hepatotoxicity [9]; and (3) had the other unknown toxic components deriving from different kava extracts [11]. Research of kava hepatotoxicity faced multiple challenges because of the numerous compounds contained in kava extracts and limited number of affected patients [61]. Therefore, more clinical and experimental studies are needed to increase the knowledge of this field, and then, the relationship between kava and hepatotoxicitye can be elucidated in the future [70].

A number of studies have reported the anticancer activity of kava extraction or the isolated individual components. The most investigated compound of kava was found to be flavokavain B followed by flavokavain A, which all belong to the chalcone family but possess different substituents on their aryl rings. The biological activities of chalcones were associated with the presence of a double bond in conjugation with carbonyl functionality [95, 96]. The mechanism of antiproliferative effect of kava was believed to be related with cell cycle arrest, induced apoptosis [97], and autophagy [42]. However, the role of autophagy was complex during the cancer therapy. As induced autophagy through the bioactive constituents of kava might become an attractive approach for cancer prevention and therapy in the future [44, 48], more investigations are required to identify the mechanism involved in this process. Meanwhile, the anticancer activity of kava was mainly focused on *in vitro* assessment, and only parts of studies were performed using *in vivo* models; current evidence from numerous clinical trials suggested the plant of kava was not sufficient to perform effective treatment for GAD. Therefore, future studies should be designed to fulfill these gaps. In order to give further information on the development of a new anticancer drug, more research is needed in the area of kava toxicity to explore the mechanisms of action on treat cancers, in the investigation of kava structure-activity relationship, and in the metabolism of kava. In summary, more clinical trials are needed to assess the effect of kava for treating GAD and the efficacy of kavalactones and flavokavains in treating cancers, and rational establishment of kava quality specifications will be beneficial for the general usages of kava. These reviews highlight areas for further research of kava constituents in the prevention and treatment of clinical diseases.

## Figures and Tables

**Table 1:** The pharmacological activities of kavalactones and flavokavins in kava.

S. no.	Activity/disease	Active molecule(s)	Model system	Methods/dosage	Result or major finding	Reference
**1**	Sedative property	Kavain	Male Wistar rats	*In vivo* Kavain 10, 30, and 100 mg/kg, p.o. suspended in 0.5% carboxymethyl cellulose solution	(i) Shortened the sleep latency with kavain at doses of 30 and 100 mg/kg (ii) Decreased the awake time with kavain at a dose of 3 mg/kg (iii) Increase the nonrapid eye movement(non-REM) sleep time with kavain at doses of 30 and 100 mg/kg (iv) No significant effects in total REM sleep time with kavain at any used doses (v) Increased the delta activity during non-REM sleep using kavain at doses of 30 and 100 mg/kg	[23]

**2**	Anti-inflammatory activity	Kavain	Mouse macrophages, mouse bone marrow macrophages (BMM), leukemia cells in mouse macrophage (RAW 264.7 cells), THP-1 cells, and human peripheral blood mononuclear cells (HPBMC); wild-type (WT) mice	*In vitro* 200 *μ*g/ml kavain. Western blot analysis. *In vivo*, 4 mg/kg. Enzyme-linked immunosorbent assay (ELISA)	(i) *In vitro*, kavain reduced lipopolysaccharide- (LPS-) induced tumor necrosis factor-*α* (TNF-*α*) secretion in BMM and HPBMC (ii) Kavain treatment in RAW264.7 cells deactivated myeloid differentiation factor 88(MyD88), inhibited lipoplysaccharide-induced TNF-a activating factor (LITAF), and reduced the production of TNF-*α*, (interleukin) IL-27 and membrane immunoglobulin (MIG) in response to LPS (iii) *In vivo*, kavain showed a significant anti-inflammatory effect on wild-type (WT) mice that developed collagen antibody-induced arthritis (CAIA)	[24]
		Kavain analogue (Kav001)	Mouse bone marrow macrophages (BMM) and THP-1 cells; wild-type (WT) mice	*In vitro* and *in vivo* Enzyme-linked immunosorbent assay, endotoxic shock assay, western blot analysis, and cytotoxicity tests	(i) Kav001 significantly inhibited *P. gingivalis*-induced CAIA/endotoxic shock (ii) Kav001-treated mice or macrophages quickly initiated their immune system to protect the host (mouse or cells) from *P. gingivalis* and LPS-induced TNF-*α* secretion via induction of B-cell lymphoma 6 (Bcl-6) and reduction of LITAF expression	[25]
		Flavokawain A (FKA)	RAW 264.7 cells	*In vitro* Western blot analysis, enzyme-linked immunosorbent assay (ELISA), electrophoretic mobility shift assay (EMSA), and transient transfection and luciferase assay	(i) Flavokawain A inhibited inducible NO synthase (iNOS) and cyclooxygenase (COX-2) expression and subsequent production of NO and prostaglandin E2 (PGE2) (ii) Flavokawain A inhibited LPS-induced NF-kB and amphipathic protein 1 (AP-1) activation (iii) Flavokawain A inhibited the production of proinflammatory cytokines, such as TNF-*α*, interleukin-1*β*(IL-1*β*), and IL-6	[26]
	Periodontitis	Kava-241 compound (kavain analogue)	RAW 264.7 cells	Kava-241 40 mg/kg. *In vitro,* enzyme-linked immunosorbent assay (ELISA) and cytotoxicity assay	(i) Kava-241 treatment was associated to reduced cell death than kava treatment (*p* < 0.05) (ii) Both kava-241 treatment and prevention reduced alveolar bone loss (by 36.98% and 39.05%, respectively)	[27]
	Rheumatoid arthritis (RA)	Kava-241 compound (kavain analogue)	Pathogen-free DBA1/BO male mice	Kava-241 40 mg/kg *In vivo* Western blot analysis, enzyme-linked immunosorbent assay (ELISA), clinical inflammation score, and radiological analysis	(i) Kava-241 reduced inflammatory cells recruitment and osteoclast activation (ii) Kava-241 treatment of *P. gingivalis*-infected BMMs reduced TNF-*α* secretion in a dose-dependent manner (40% decrease for 20 *μ*g/ml, 70% for 100 *μ*g/ml, and 90% for 200 *μ*g/ml)	[28]

**3**	Colon cancer	Flavokawain C (FKC)	Human colon adenocarcinoma HT-29 and human carcinoma HCT 116 cells	*In vitro* Sulforhodamine B assay, dichlorofluorescein fluorescence staining, spectrophotometric method, and western blot analysis	(i) FKC-induced G2/M arrest upregulated the cyclin kinase inhibitor proteins (p21 and p27) independent of p53 (ii) FKC induced apoptosis via activation of caspase- 3, -8, and -9 (iii) FKC increased the reactive oxygen species (ROS) generation and reduced the superoxide dismutase) (SOD) activity (iv) FKC triggered the endoplasmic reticulum (ER) stress-mediated apoptosis and inactivated inhibitor of apoptosis proteins (IAPs)	[29]
		Flavokawain B (FKB)	Human colorectal adenocarcinoma cell line LoVo and its doxorubicin-resistant subline LoVo/Dx	*In vitro* Sulforhodamine assay, western blotting and immunodetection, and flow cytometry measurements	(i) The ability of FKB to inhibit the proliferation in both cell lines was detected (ii) FKB induced cell cycle arrest in the G2/M phase and the presence of SubG1 fraction and induced apoptosis (iii) Flavokawain B at low concentration led to increase of caspase-3 activity	[30]
		Dihydromethysticin (DHM)	Colorectal cancer (CRC) cell lines (HCT116, HT29, and LoVo). Normal human colonic mucosal epithelial cells (NCM460)	*In vitro* and *in vivo,* cell viability assay, transwell invasion assay, wound healing assay, cell apoptosis assay, terminal dUTP nick-end labeling assay and 4′,6-diamidino-2-phenylindole staining, and western blot analysis	(i) DHM inhibited CRC cell proliferation, invasion, and migration (ii) DHM induced apoptosis by upregulating cleaved caspase-3 and B-cell lymphoma 2 (Bcl-2) expression and downregulating BCL2-associated X(Bax) expression (iii) DHM induced *G*0/*G*1 phase arrest by regulating cyclin D1 and cyclin-dependent kinase 4 (CDK4) expression (iv) DHM restricted CRC tumor growth *in vivo* partially by altering the NLRC3/PI3K pathway	[31]
	Lung cancer	Flavokawain B (FKB)	Human lung cancer cell line NSCLC H460	*In vitro* Methyl thiazolyl tetrazolium (MTT) assay, cell morphology observation fluorescence-activated cell sorting, and western blot analysis	(i) FKB inhibited the cell proliferation via inducing G2-M cell cycle arrest and apoptosis in H460 cells (ii) FKB treatment resulted in cytochrome c release and activated the cleavage of poly ADP ribose polymerase (PARP), caspase-7, and caspase-9 (iii) FKB induced apoptosis of H460 cells through the Bax-initiated mitochondrial pathway and Jun N-terminal kinase (JNK) pathway	[32]
		Kava and kavalactones	NCI-H1299 cells	*In vitro* Detection of intracellular calcium influx	(i) Kava extract effectively inhibited norepinephrine- (NE-) mediated intracellular calcium influx potentially through antagonizing *β*-adrenergic receptor (*β*-AR) signaling (ii) The overall potency rank of these 6 major kavalactones in inhibiting NE-induced calcium responses are as follows: DHK, Y, K, DMY, M, and DHM	[33]
		Dihydromethysticin (DHM)	C57BL/6 female mice	*In vivo* Mouse liver microsome preparation and CYP1A1/2 enzymatic assay (EROD), western blot analysis, and quantitative reverse transcription polymerase chain reaction (PCR)	(i) Reduction in the level of O^6^-methylguanine(O^6^-mg) by DHM was AhR independent (ii) Smaller doses of DHM may be sufficient to enhance NNAL glucuronidation in the target lung tissue (ii) DHM at 0.2 or 0.05 mg/g of diet, while retaining the complete chemopreventive effect, did not cause any induction of CYP1A1/2 activity in the liver microsome	[34]
	Osteosarcoma (OS)	Dihydromethysticin (DHM)	Human osteosarcoma cell line (MG-63)	*In vitro* MTT assay, V-FITC assay, flow cytometry analysis, fluorescence microscopy, video microscopy, immunoblotting analysis, and western blot analysis	(i) Dihydromethysticin induced dose-dependent as well as time-dependent antiproliferative effects against MG-63 cell growth (ii) DHM induced an increase in *G*0/*G*1 cells (iii) Dihydromethysticin treatment induced mitochondrial transmembrane depolarization and decreased phosphorylation levels for phosphatidylinositol-3-hydroxykinase (PI3K), AKT (Ser 473), and glycogen synthase kinase (GSK-3*β*)	[35]
		Flavokavain B (FKB)	OS160 cell, human OS cell lines. 143B, SaOS-2, MG-63, and U2OS	*In vitro* MTT assay, soft agar colony formation assay, DAPI staining, FACS analysis, and western blot analysis	(i) FKB induced apoptosis via increasing the expression of Fas, Puma, and Bax and downregulating the expression of Bcl-2 and survivin (ii) FKB treatment increased caspase 8, 9, and 3/7 activity and inhibited the secretion of both matrix metalloproteinases (MMPs) in a dose-dependent manner (iii) FKB induced G2/M phase cell cycle arrest via decreasing the levels of cdc2 and Cyclin B1 and increasing the levels of myelin transcription factor 1 (Myt 1)	[36]
		Flavokavain A (FKA)	Standard human osteosarcoma cell lines 143B, SaOS-2, HOS, and U2OS, metastatic cell line SaOS-LM7, and patient-derived osteosarcoma cell line	*In vivo* and *in vitro* Quantitative RT-PCR assay, fluorescence-activated cell sorting (FACS) analysis, immunohistochemistry (IHC), and western blot analysis	(i) Genetic knockdown of S phase kinase-associated protein (Skp2) reduced osteosarcoma proliferation and invasion (ii) Genetic knockdown of Skp2 reduced osteosarcoma growth and metastasis *in vivo* (iii) FKA decreased Skp2 expression in osteosarcoma cells (iv) Oral treatment with FKA inhibited osteosarcoma lung metastasis *in vivo*	[37]
	Synovial sarcomas (SS)	Flavokawain B (FKB)	SS cell lines SYO-I and HS-SY-II	*In vitro* MTT assay, western blot analysis, and RT-PCR assay	(i) FKB induced apoptosis by the activation of caspase-3/7, -8, and -9, upregulating the expression of proapoptotic markers, and downregulating antiapoptotic marker expression	[38]
	Breast cancer	FlavokavainA (FKA)	Cell lines Michigan cancer foundation (MCF)-7, MDA-MB231, and MCF-10A	*In vitro* MTT assay, AO/PI double staining, annexin V/FITC assay, quantitative RT-PCR assay, and western blot analysis	(i) Flavokawain A induced apoptosis through activating caspase-8 and -9 (ii) FKA possessed antiangiogenic potential (iii) FKA regulated several apoptosis and metastatic-related genes and proteins	[39]
		Flavokavain B (FKB)	Cell lines MCF-7, MDA-MB231, and MCF-10A	*In vitro* MTT assay, BrdU incorporation assay, annexin V/FITC assay, Proteome profiler array ^TM^, quantitative RT-PCR assay, and western blot analysis	(i) Flavokawain B induced G2/M arrest and apoptosis in MDA-MB231 and MCF-7 (ii) Flavokawain B inhibited migration and invasion *in vitro* and suppressed the formation of tube-like vessels *in vitro* (iii) Flavokawain B regulated several metastasis-related proteins and genes and tyrosine kinases in MDA-MB231	[40]
	Thyroid cancer (TCa)	Flavokavain B (FKB)	Human TCa cell lines ARO, WRO, and TPC-1; athymic mice	*In vitro* and *in vivo,* flow cytometric analysis, western blotting, transmission electron microscopy, and immunohistochemistry	(i) Flavokawain B induced mitochondrial dysfunction in TCa cells (ii) Flavokawain B induced autophagy by inhibiting the mammalian target of rapamyoin (mTOR) pathway and activating beclin-1 and AMP-activated protein kinase (AMPK) in TCa cells (iii) Flavokawain B induced cytoprotective autophagy in TCa cells both *in vitro* and *in vivo*	[41]
	Gastric cancer	Flavokavain B (FKB)	Human gastric adenocarcinoma (AGS), gastric carcinoma NCI-N87, Kato-III, mixed cells (stomach and intestine) Hs738, and human gastric cancer cell line TSGH 9201; female athymic nude mice	*In vitro* and *in vivo* MTT assay, western blot analysis, and acridine orange staining	(i) FKB induced both autophagy and apoptosis in gastric cancer NCI-N87 cells (ii) FKB causes G2/M arrest through reactive oxygen species (ROS) c-jun N-terminal kinase (JNK) signaling pathways in AGS cells (iii) FKB suppressed the human epidermal growth factor receptor 2 (HER-2) expression and PI3K/Akt/mTOR signaling pathways through the induction of autophagy in human gastric cancer cells (iii) FKB inhibited AGS tumor development through the induction of an autophagic mechanism *in vivo*	[42]
	Bladder cancer	Flavokavain A (FKA)	Low copy male and female transgenic mice UPII-SV40 T	*In vivo,* immunohistochemistry, DeadEnd colorimetric TUNEL assay, and western blotting	(i) FKA feeding increased the survival of male low-copy UPII-SV40 T transgenic mice and reduced the weight of tumor-bearing urinary bladders (ii) FKA feeding decreased proliferation and increased apoptosis in bladder tissues (iii) FKA feeding affected the expression of apoptosis and cell-cycle regulators	[43]
		Yangonin	RT4, T24, UMUC3, and HT 1193 cell lines	*In vitro,* MTT assay, colony formation assay, western blot analysis, fluorescence microscopy, and electron microscopy	(i) Yangonin induced autophagy through the inhibition of the mTOR pathway, increasing the expression of beclin, ATG5, and LKB1, and decreasing the phosphorylation of Akt, PRAS40, rpS6, p70S6K, and 4E-BP1 (ii) Yangonin inhibited the development and progression of bladder cancer synergistically with docetaxel and flavokawain A	[44]
	Prostate cancer (PCa)	Flavokavain B (FKB)	PCa cell lines LNCaP, PC3, and C4-2B	*In vitro,* MTT assay and western blot analysis	(i) FKB inhibited Cullin-1 and Ubc12 neddylation in LNCaP and PC3 cells (ii) FKB interacted with the NAE1 regulatory subunit to inhibit UBC12 neddylation (ii) Flavokavain B-induced Skp2 degradation was dependent on functional Cullin-1 via increasing Skp2 ubiquitination	[45]
		Flavokavain A (FKA)	PC3 cell line	*In vitro* Immunofluorescent analysis, western blotting, and metabolomics assays	(i) FKA induced PC3 cell cycle arrest by regulating the expression of survivin proteins (ii) FKA was active against tubulin polymerization (iii) Major metabolic pathways that were changed after FKA treatment included sphingolipid metabolism, biosynthesis of fatty acids, metabolism of D-glutamine and D-glutamic acid, metabolism of alanine, aspartic acid and glutamic acid, and glutathione metabolism	[46]
		Kavalactone-rich kava fraction	Male C57BL/6J and female C57BL/6-Tg TRAMP 8247Ng/J mice	*In vivo* Histology and immunohistochemical (IHC) staining and real-time qRT-PCR	(i) Dietary KFB consumption decreased the incidence of neuroendocrine carcinomas (NECa) (ii) Dietary KFB consumption increased relative liver weight without affecting hepatic integrity (iii) KFB diet consumption suppressed the growth of transgenic adenocarcinoma of mouse prostate (TRAMP) epithelial lesions and modified a spectrum of genes in the TRAMP dorsolateral prostate (DLP) on the one hand	[47]
	Glioblastoma multiforme (GBM)	Flavokawain B (FKB)	Human glioma cell lines U251 and U87, fibroblast glioblastoma cell line T98, GBM biopsy xenograft propagated tumor cells P3, and luciferase-stable U251 glioma cells	*In vitro* and *in vivo,* immunofluorescence staining, transmission electron microscopy (TEM), western blot analysis, immunohistochemistry, and TUNEL assay	(i) FKB inhibited the proliferation of GBM cells *in vitro* (ii) FKB induced cellular senescence and autophagy in GBM cells *in vitro* (iii) FKB induced autophagy through ER stress-dependent upregulation of activating transcription factor 4(ATF4) and DNA damage inducible transcript 3(DDIT3) and the ATF4-DDIT3 (tribbles pseudokinase 3) TRIB3-AKT-MTOR-RPS6KB1 signaling pathway in GBM cells (iv) FKB inhibited the growth of GBM cells *in vivo*	[48]
	Uterine leiomyosarcoma	Flavokawain B (FKB)	SK-LMS-1, ECC-1(endometrial adenocarcinoma), and T-HESC (normal endometrial fibroblasts) cell lines	*In vitro,* MTT assay, FACS analysis, western blot analysis, and RT-PCR	(i) FKB induces apoptosis and G2/M arrest in SK-LMS-1 and ECC-1 cells (ii) FKB induced apoptosis through upregulating the expression of proapoptotic proteins and downregulating survivin expression (iii) FKB acted synergistically with gemcitabine and docetaxel in inhibiting the growth of SK-LMS-1 cells	[49]
	Oral cancer	Flavokawain B (FKB)	Human oral squamous carcinoma HSC-3, melanoma A-2058, adenosquamous carcinoma Cal-27, and lung carcinoma A-549 cells	*In vitro* Immunofluorescence assay and western blot analysis	(i) FKB inhibited G2/M cell-cycle arrest through reducing the levels of cyclin A, cyclin B1, Cdc2, and Cdc25 C in HSC-3 cells (ii) FKB-induced apoptosis was mediated by both caspase-dependent and caspase-independent mechanisms (iii) FKB-mediated inactivation of the PI3K/Akt and p38 mitogen-activated protein kinase (MAPK) signaling pathway played a functional role in G2/M arrest and apoptosis in HSC-3 cells (iv) FKB treatment induced an early increase in intracellular ROS generation	[50]
	Oral adenoid cystic carcinoma (ACC)	Flavokawain B (FKB)	ACC-2 cell line	*In vitro* MTT assay, DAPI staining, RT-PCR fluorescence-activated cell sorting analysis, and western blot analysis	(i) FKB significantly inhibited the cell proliferation of ACC-2 in a dose-dependent manner (ii) The IC50 of flavokawain B treatment for 48 h was estimated to be 4.69 ± 0.43 micromol/L (iii) Flavokawain B induced apoptosis and cell cycle G2-M arrests in ACC-2 cells (iv) FKB might be through the induction of apoptosis in Bax-initiated mitochondrial pathways and caspase-3-dependent cellular apoptotic pathways (v) Flavokawain B differentially induced the mRNA expression of Bim, Bak, Bax, and Bcl-2	[51]
	Squamous carcinoma	Flavokawain B (FKB)	Human squamous carcinoma cell line KB and human gingival fibroblast cell line HGF; female athymic nude mice (BALB/c-nu)	*In vitro* and *in vivo,* terminal deoxynucleotidyl transferase-meditated dUTP nick end labeling assay, flow cytometric analysis, and western blotting	(i) FKB induced apoptotic DNA fragmentation (ii) FKB induced the release of cytochrome c and activation of caspase-3 and -9 and cleavage of PARP (iii) FKB induced dysregulation of Bcl-2 and Bax proteins (iv) FKB inhibited G2/M cell cycle arrest by reducing the levels of cyclin A, cyclin B1, Cdc2, and Cdc25 C (v) FKB inhibited KB xenograft growth *in vivo*	[52]
	Acute lymphoblastic leukemia (ALL)	Flavokawain B (FKB)	HK-2 (normal renal proximal tubular cell), CCRF- CEM (T-ALL), CEM-C1 (T-ALL), Jurkat (T-ALL), and RS4-11 (B-ALL) cell lines. 11 patients with B-ALL, five patients with T-ALL, and four normal volunteers; female Balb/c mice	*In vitro* and *in vivo* Western blot assay	(i) Flavokawain B inhibited the proliferation of various types of ALL cell lines (ii) Flavokawain B induced apoptosis through increasing caspase-3 activity and PARP cleavage and promoting the expression of p53, Bax, and Puma in ALL cells (iii) Flavokawain B inhibited the growth of patient-derived ALL blasts ex vivo (iv) FKB suppresses xenografted human ALL in mice	[53]
	Cervical cancer	Flavokawain B (FKB)	Cervical cancer HeLa cells	*In vitro* MTT assay, flow cytometry analyses, and qRT-PCR	(i) FKB induced cytotoxicity against HeLa cells (ii) FKB induced G2/M phase arrest and apoptosis through the loss of membrane potential (iii) FKB induced cell death through p21-mediated cell cycle arrest and activation of p38 (iv) FKB treatment enhanced the GSH and SOD levels in HeLa cells (v) FKB protected HeLa cells from H_2_O_2_-induced cell death via neutralization of reactive oxygen species (ROS) (vi) FKB failed to induce apoptosis in HeLa cells via oxidative stress	[54]
	Antiangiogenic activity	Flavokawain B (FKB)	Human umbilical vein endothelial cells (HUVECs) and human brain endothelial cells zebrafish strain	*In vitro* and *in vivo* MTT assay and tube formation assay	(i) FKB inhibited endothelial cell proliferation, migration, and tube formation even at very low and nontoxic concentrations (ii) FKB blocked the angiogenesis process in zebrafish with a dramatic reduction of subintestinal vein formation in a dose-dependent manner (iii) FKB at the concentration of 2.5 *μ*g/mL did not exhibit any toxic effects in zebrafish larvae and caused a marked or complete obliteration of subintestinal vein formation	[55]

**4**	Antifibrotic and antioxidant properties	Flavokavain A (FKA)	Rat aortic smooth muscle cell line (A7r5)	*In vitro* MTT assay, western blot analysis, and immunofluorescence assay	(i) FKA was not cytotoxic for A7r5 cells without transforming growth factor (TGF)-*β*1 stimulation (ii) FKA suppressed TGF-*β*1-induced fibrosis via the inhibition of F-actin, *α*‐smooth muscle actin(*α*-SMA), and fibronectin (iii) FKA pretreatment inhibits TGF-*β*1-induced migration and invasion (iv) FKA potentiated nuclear factor E2-related factor 2(Nrf2) activation and nuclear translocation in A7r5 cells (v) Nrf2 knockdown diminished FKA-induced antioxidant responses in A7r5 cells	[56]
	Immunomodulatory property	Flavokavain A (FKA) and flavokawain B (FKB)	Male BALB/c mice	*In vitro* and *in vivo* MTT assay and serum biochemical analysis	(i) FKA and FKB did not cause mortality, and all mice were observed normal after the treatment period (ii) FKA and FKB may have the potential as an immunomodulatory agent (iii) FKA and FKB did not significantly alter the body weight and serum biochemical profile of the mice	[57]

## Data Availability

No data were used to support this study.

## Abbreviations

ACC: Adenoid cystic carcinoma
ALL: Acute lymphoblastic leukemia
BMM: Bone marrow macrophages
COX: Cyclooxygenase
CRC: Colorectal cancer
DHM: Dihydromethysticin
ELISA: Enzyme-linked immunosorbent assay
FKA: Flavokawain A
FKB: Flavokawain B
FKC: Flavokawain C
GAD: Generalized anxiety disorder
HPLC: High-performance liquid chromatography
LPS: Lipopolysaccharide
MCF: Michigan cancer foundation
MTT: Methyl thiazolyl tetrazolium
NF-*κ*B: Nuclear factor-*κ*B
PCR: Polymerase chain reaction
PI3K: Phosphoinositide 3-kinase
Skp2: S phase kinase-associated protein 2
TCa: Thyroid cancer
TGF: Transforming growth factor
TNF-*α*: Tumor necrosis factor-*α*
TRAMP: Transgenic adenocarcinoma of the mouse prostate
WT: Wild type.
